# Polymyositis

**Published:** 1899-01-28

**Authors:** 


### POLYMYOSITIS.

In a lecture delivered at the National Hospital for the Paralysed and Epileptic Sir William Gowers[1] has drawn attention to the disease known as Polymyositis, which consists in the simultaneous inflammation of many muscles, involving often the inflammation of some of the nerves supplying them, and leading to a widespread form of paralysis. One essential and striking feature of the disease is that it is symmetrical, affecting both sides of the body alike. Not that the symmetry is absolute, but the correspondence of the two sides is the dominant feature, and they seldom present conspicuous differences either in the area involved or in the symptoms, thus reminding one much of the simi-

[1] Brit. Med. Jour., Jan. 14.

larly symmetrical distribution of certain forms of peripheral neuritis.

This distribution brings one to the cause. For any agent to pick out and act upon parts of the body so distant from each other as are the corresponding parts of the two sides of the body it must reach them by the only path by which they are equally accessible, namely, the circulation. True it is, no doubt, that from inherent defect of vitality certain tissues of the body may tend to fail sooner than other tissues, and that from the symmetrical distribution of these feeble structures they may, when they give way, produce an appearance of symmetrical disease, but in such cases the failure is generally a senile change, and in any case is an extremely gradual process, very unlike the comparatively rapid development of the disease in question. We must, then, take bilateral symmetry of symptoms as indicating that the cause is some toxic agent in the blood. Sometimes this may be something received from without, as, for example, metallic poisons or alcohol, often it is something made within the body, such as a toxin arising from bacterial growth, as in diphtheria and other specific diseases. But toxins may be produced by mere alterations of the normal metabolism of the body. Most complex chemical changes are constantly going on in our bodies, a very slight disturbance of which—such, for example, as may be set up by mere exposure to cold—may lead to the development of toxic products, which in turn may cause the symptoms which we call disease; and such toxins, when they have special affinities for special tissues which are symmetrically distributed, produce symptoms which are themselves symmetrical.

The patient who formed the text of Sir William Gowers' address was a woman aged 36, the wife of a labourer. Her father had suffered twice from rheumatic fever, and his father again had suffered from gout. The first symptoms of which the patient complained were a constant tired feeling and aching pain in the back and around the loins. These came on after prolonged over-fatigue and exposure to cold at night. Then an irritable rash appeared on the hands and arms, and she had pain in the hands and ankles, and both hands and feet became weak. Gradually her feet dropped so that she could not get upstairs, she had difficulty in sitting upright, the limbs and trunk became first weak then stiff, so that she could neither stand nor bend forwards. Then while the pain gradually lessened the stiffness increased and the arms and legs became rigid in the position of flexion, till in about a year and a half she was reduced to a condition of almost universal palsy. When admitted she was so helpless that she could not sit up. The facial muscles acted slowly and imperfectly, apparently from stiffness, and there was contraction of the masseters, which prevented the mouth from being widely opened. There was rigid contraction of all the muscles of the neck, causing the head to be quite stiff upon the trunk. The same rigidity existed in the muscles of the back, so that she could not bend the trunk either backwards or forwards, and had to be moved in bed as one stiff piece. The same condition of rigidity and contraction was most marked in all the muscles of the upper limbs, although slight voluntary contraction could be produced in most of them. The muscles were much wasted, but, although small, they were extremely hard and firm. They reacted very slightly to the different forms of electricity, but such response as there was to the interrupted voltaic current was of the sharp, quick character which indicates stimulation of intramuscular nerves, and not of the muscular substance itself. Both legs were rigid and flexed, and the muscles were small and hard. She had, however, just power left in the flexors of the hip to raise each foot from the bed. Sensibility in both arms and legs was normal, and no pain or subjective sensation was complained of except pain in the back and shoulders. A curious symptom was the excessive perspiration both on the face and the limbs. Apart from exertion, the sweat would often stand in beads on the surface.

Now, it must be recognised that when this condition had been reached, all hope of cure was gone. The inflammatory process in the muscles, which had been the essence of the disease, had attained its cicatricial stage, and the newly-formed connective tissue, almost universally distributed, had already contracted. Many muscular fibres had perished in consequence of the acute inflammation and of the degeneration of their nerve fibres; and although it was possible that by exercise some increase of power might be hoped for in such as remained, the contracture of the interspersed cicatricial fibres placed insuperable obstacles in the way of anything like complete recovery.

The moment for really effective treatment in such cases as this is in the early period of the case when the malady is in the stage of development. In the stage of contraction we have to deal not with the disease, but with its results. But when inflammation of the muscles is actually going on under the influence of toxins present in the blood much can be done, and it can hardly be doubted that rest, diaphoretics, salicylates, with perhaps small doses of mercury, would in that stage have controlled the inflammation, and might have very much lessened the mischief to which it subsequently led.

A highly specialised tissue, such as muscular tissue is, cannot remain inflamed for long with impunity, and a toxic myositis, such as results from exposure to cold, must be treated early if contraction is to be prevented.

